# The Triple-S framework: ensuring scalable, sustainable, and serviceable practices in educational technology

**DOI:** 10.1186/s41239-022-00378-y

**Published:** 2023-02-13

**Authors:** Christian Moro, Kathy A. Mills, Charlotte Phelps, James Birt

**Affiliations:** 1 Faculty of Health Sciences and Medicine, Bond University, Gold Coast, QLD 4226 Australia; 2 Institute for Learning Sciences and Teacher Education, Australian Catholic University, Brisbane, QLD 4000 Australia; 3 Faculty of Society and Design, Bond University, Gold Coast, QLD 4226 Australia

**Keywords:** Technology-enhanced learning, Tertiary education, Blended learning, Sustainability, e-learning

## Abstract

Educational institutions are increasingly investing into digital delivery, acquiring new devices, and employing novel software and services. The rising costs associated with maintenance, in combination with increasing redundancy of older technologies, presents multiple challenges. While lesson content itself may not have changed, the educational landscape constantly evolves, where tertiary institutions are incorporating new modes of content delivery, hybrid-style learning, and interactive technologies. Investments into digital expansions must be taken with caution, particularly prior to the procurement of technology, with a need for the proposed interventions’ scalability, sustainability, and serviceability to be considered. This article presents the Triple-S framework for educators, administrators, and educational institutions, and outlines examples of its application within curricula. The paper synthesises research evidence to provide the foundation underlying the key principles of the Triple-S framework, presenting a useful model to use when evaluating digital interventions. Utilising the framework for decisions regarding the acquisition of educational technology, devices, software, applications, and online resources can assist in the assurance of viable and  appropriate investments.

**Graphical Abstract:**

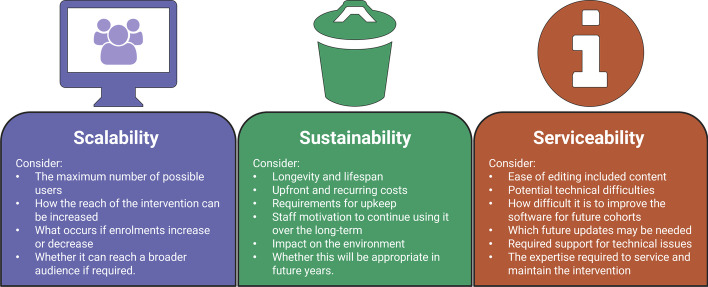

## Introduction

Education is changing in the context of rapidly shifting communication media and a multiplicity of modes (Cope & Kalantzis, 1999), requiring new tools to evaluate the adoption of digital technologies for learning. In many cases, educators might feel compelled to introduce new and novel content delivery modes in the curricula. However, in doing so, educators should be aware that different technologies have varied complexities in their ability to be scalable, sustainable, or serviceable.

This article proposes the use of the Triple-S framework, an original research-based model for assessing proposed technological reforms and digital adoption in classrooms and organisations. The framework can be applied to decision-making about educational investment in new hardware, software, technology interventions, or curriculum-based digital reform, to systematically consider scalability, sustainability, and serviceability. Scalability is defined as the ability to handle increased workload without adding resources to a system (Weinstock & Goodenough, 2006). Sustainability refers to the quality of being able to continue over a period of time (Moore et al., 2017), and also encompasses environmental sustainability, which is the quality of causing little or no damage to the environment, and therefore, able to continue for a long time. Serviceability denotes the speed, courtesy, competence, and ease of repair of an intervention (Garvin, 1987). The use of the Triple-S framework in educational settings aims to inhibit ‘innovation for the sake of innovation’ (Murtha-Lemekhova et al., 2022), and encourages educators and educational organisational decision-makers to assess the gains and losses of any new digital technology intervention on each aspect of the Triple-S framework.

### Forming the Triple-S framework

For many years, it has been recognised that information technology (IT) has the potential to revolutionise the fundamental business of teaching and learning in the classroom (Chandra & Mills, 2015), but universities are often resistant to change and can be risk-averse (Kaplan, 2021). The 2022 EDUCAUSE Horizon Report identified this risk aversion, with ‘cost’ emerging as the most significant obstacle to technology adoption in education (Pelletier et al., 2022). This concern perhaps is justified with global IT funds for teaching and learning decreasing (Grajek, 2020), as IT expenses grow and finances are spread thin across multiple priorities, such as student learning achievement, student and staff safety and health, meeting enrolment objectives, and a move to online instruction during COVID-19. In addition, priorities of institutional teaching and learning and IT are shifting, resulting in a reduced allocation for quality education. This is compounded by an ever-increasing range of technology-enhanced options for supporting learning and their overall perceived usefulness by students.

According to a recent study by Kennedy and Dunn (2018), student participants desired their instructors to utilise more technology, but they also wanted more consistency in their use. Furthermore, they want encouraging connections with professors and fellow students via technology, as well as access to new resources and wider reading materials, implying a novel use of technology. In a recent study by Cohen et al. (2022), perceived technology usefulness also differs not only across cohorts and countries, but also across official and unofficial technology tools used by learners. In truth, students extensively employ common technology in their academic studies (Rashid & Asghar, 2016), including Internet search engines to find information, audio recordings or videos about subjects/disciplines (e.g., YouTube, Vimeo), non-university provided scholarly websites to search for papers/journals, social networking sites to collaborate with other students on courses (e.g., Facebook or Twitter), and Wikipedia to find information.

As educators, we want to encourage students to try out new tools and services, but we must also be cognisant of consistency of usage, pedagogical fit for purpose in the classroom, and cost–benefit analysis when contemplating using educational technology (Callimaci & Fortin, 2022). Callimaci and Fortin (2022) also address the ever-growing complexities of technology and its impact on educators, with educators often influenced by their co-workers' opinions and institutional directives resulting in a haphazard approach to the integration of technology. This becomes more complicated as our classrooms evolve, as witnessed during the COVID-19 pandemic, with face-to-face education being converted to emergency remote teaching—the emergency act of teaching to remote learners in pressing circumstances. Emergency teaching is distinct from high quality online learning (Hodges et al., 2020) and Hyflex delivery (flexible course design involving student choice between synchronous, face-to-face or online learning), as well as a shift away from didactic lectures further towards experiential learning (Kohnke & Moorhouse, 2021). Therefore, we need to formalise an approach.

### The Triple-S framework

**Fig. 1:**
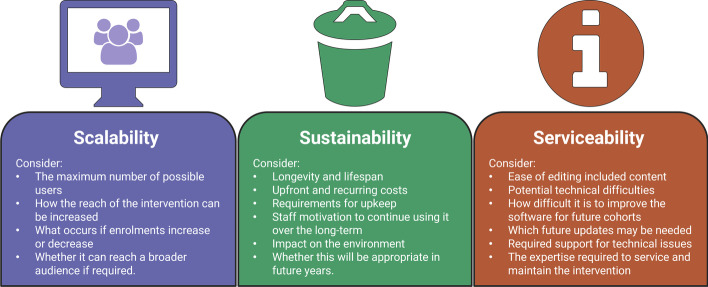
The Triple-S framework

The Triple-S framework assists to ensure Scalable, Sustainable, and Serviceable practices for digital curricula and educational technology procurement (Fig. 1).

#### Scalability

Although a wide variety of digital content can be scaled quite easily, there is a disparity between individual students regarding their ability to access technologies and the internet (Raes et al., 2020). The differences range from the quality of home broadband, to the suitability of a home environment (Cullinan et al., 2021), or the native digital literacy skills required to engage with the content (Silva et al., 2018). In fact, the technological skills required to access educational material changes so regularly (Maycock et al., 2018) that students run the risk of being left behind (Bączek et al., 2021). For example, if a new method of dissemination were to be developed, which educators can use to scale an educational intervention to a larger cohort, there will be variations in how effectively each student engages with it, and whether the technology will be distracting to the students, inhibiting the learning experience. In addition, students may not work well in isolated environments, away from their peers, and others may find that the sheer volume of digital content required to consume becomes overwhelming (Rasheed et al., 2020). This means that although appealing, the digital scaling of such educational content may not be as effective as envisioned in the long run.

During the COVID-19 pandemic, there was a considerable challenge for tertiary institutions that focussed on presence-teaching or face-to-face models of instruction. The more scalable the curricula, the easier it was for educators to provide large numbers of students with academic material to facilitate continued study (da Matta & Felisberto, 2022). This presents one additional benefit to maintaining readily scalable interventions within a program, which is to rapidly respond to changes, student enrolment alterations (Nikas et al., 2022), or larger student numbers if required.

Many of the newly introduced disruptive technologies in a modern curriculum, such as augmented and virtual reality, involve substantial investments (Iglesias-Vázquez et al., 2007; McIntosh et al., 2006; Pottle, 2019). This not only includes the investment for the acquisition of devices, but also for servicing fees, updates, and licences. Devices can break, leaving students without any ability to access learning material until it is replaced. In addition, in many cases, such as using current head-mounted displays for virtual reality, students need to be present on-campus, meaning that this is not viable for the newer, hybrid delivery style of curricula provision requirements in universities.

#### Sustainability

Sustainability refers to the quality of being able to continue over a period of time (Moore et al., 2017). If educators are planning to invest time and money into resource generation, there must be a level of expected longevity in the intervention. Technologies that require software updates, edition changes, or other alterations beyond the control of the educator could force created lessons into redundancy. These changes are unavoidable and mean that work spent on using more innovative delivery modes may not be fruitful in the long term. For an intervention to offer longevity, and hence be sustainable over the long-term, plans need to be developed that assess its viability in later years.

More broadly, the term ‘sustainability’ also refers to environmental sustainability, which is the quality of causing little or no damage to the environment and therefore, able to continue for a long time (Cambridge University, 2022). This modern expectation to base decisions on the environmental impact must not be ignored. Tertiary institutions are being called to adopt a strong sustainable strategy in their curricula (McLean & Gibbs, 2022), to ensure activities maintain and preserve natural resources while promoting social wellbeing. In many cases, educational institutions are also encouraged to find ways to meet the United Nations Global Goals, promoting sustainability as a key strategic initiative to be considered in curriculum development (Moro et al., 2022a).

Future cohorts may not be willing to purchase and dispose of devices, cables, and batteries as they require regular updating, as this contributes to the problem of e-waste (electronic waste) management, which is becoming one of the fastest growing waste streams (Forti et al., 2018). For example, it would not be feasible to think that a device that requires battery replacements every month would be viable in the long term, either on its financial cost, or the cost to the environment (Shittu et al., 2021). Educational technologies that generate a substantial amount of e-waste will likely fall short of environmental guidelines, which are becoming increasingly strict on institutions. It will also fail to meet student expectations regarding how the educational institution responds to issues regarding sustainability (Roca-Barcelo et al., 2021).

#### Serviceability

Serviceability is defined as the *speed, courtesy, competence, and ease of repair* (Garvin, 1987), and throughout this framework refers to the teacher or education provider’s ability to maintain the technological intervention. For example, if content requires updating on a website or presentation slides, this can be readily revised to include any new information. However, commercial devices such as virtual reality headsets with multiple versions (e.g., Oculus DK2, CV1, Rift, Quest) are regularly changing with new and improved versions of software. As a result, support from the developing companies is withdrawn from older models, leading to the technology slowing down.

In some cases, universities are outsourcing the development of lesson content, for example, having an external company create a virtual reality lesson for their students (Hira et al., 2007). In this case, the source code is usually inaccessible by the institution and there is no opportunity to edit or refine the lessons over the year without going through the development company. Alternatively, even when developed internally, staff departures and changes to roles can mean that the expertise required is no longer on-hand for updates and edits. This maintenance is usually a requirement, not an option. When security patches or other updates are distributed, the new software may inhibit features relied upon by the original development design. As such, without regular updating and coding, lessons using advanced technology are unlikely to be viable for long-term use. For technology, this nearly always comes with requirements for monetary and staffing costs to be considered.

Considering serviceability will become increasingly more important as the cost of providing technology increases. It appears that in many cases, the investment in teaching and learning technology sits at an acceptable point, meeting expectations in some programs (Harrison et al., 2019). However, it is likely that future educators using more unique or non-standard online learning modules may need to justify recurring costs for the use of their desired technological intervention—something which can be challenging to perform (Krotov & Ives, 2016). Part of this justification should always include the long-term potential use of technology, which is highly correlated with its ability to be overseen, serviced, updated, and maintained. While it may be expected that once universities return to more normal teaching modes after the pandemic, it is likely that educators will aim to keep current technology, while also engaging in face-to-face delivery (Callimaci & Fortin, 2022), presenting increasing complexities in the need for information support staff to manage.

**Fig. 2:**
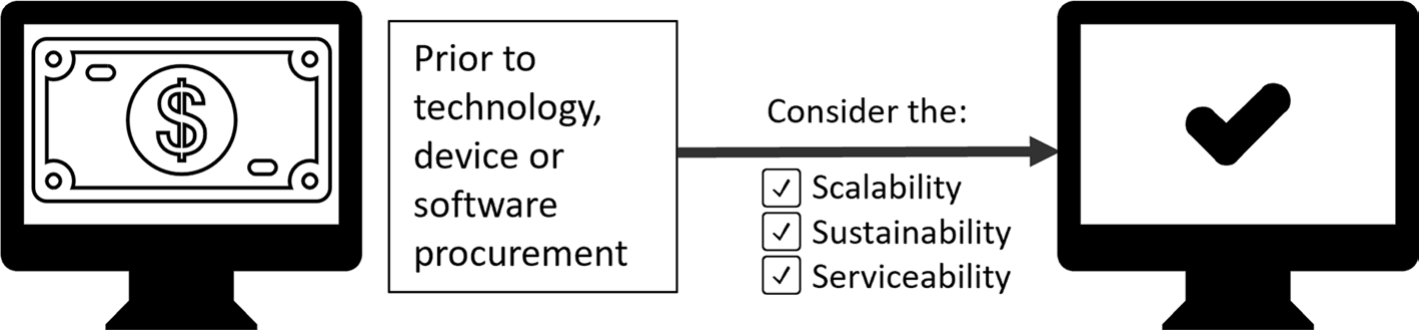
Checklist of considerations: scalability, sustainability, and serviceability

Figure 2 outlines the expected checklist for digital curricula and educational technology procurement.

### Applying the Triple-S framework: an assessment and comparative evaluation of common media examples

**Fig. 3:**
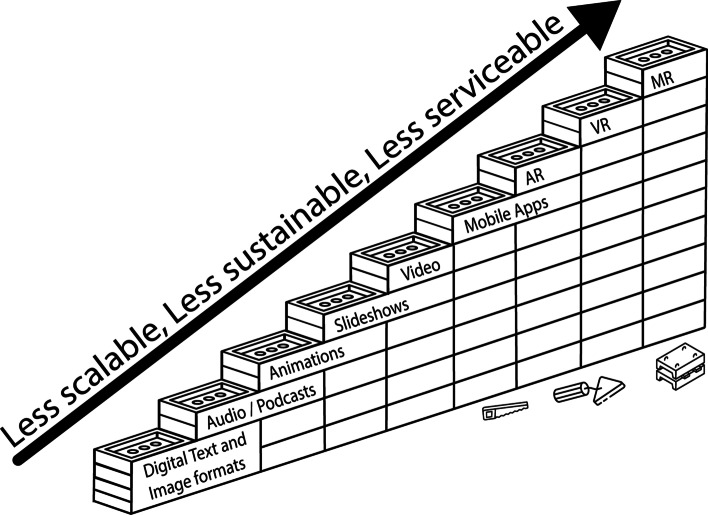
A comparative evaluation of various media examples ranked by their relative position based upon the scalability, sustainability, and serviceability

In the examples provided below, the Triple-S framework has been applied to assess the scalability, sustainability, and serviceability of some common media. As the examples progress in complexity, they tend to ‘build upon’ each other (Fig. 3). For example, text and images form a commonly used foundation, with audio, animations, and videos following. However, each media has a different level of scalability, sustainability, and serviceability. As such, the list below provides some use cases for the Triple-S framework with a comparative evaluation of common media.

#### Digital text and image formats

Digital text and image formats are the most scalable, sustainable, and serviceable delivery mode for disseminating content in an academic setting, with many common text files accessible to most users via smartphones, tablets, and computers (e.g., txt,.doc,.docx,.rtf,.log,.tex). Up-to-date text with accompanying visual information can be immediately dispersed to students anywhere in the world, particularly since the emergence of the internet and the rise of web 2.0—the read–write web and social media (Mills, 2015b). This is highly accessible to students and faculty, and using text can ensure that curricula remain updated, relevant, and modern, often without any technical expertise or proprietary software (e.g., plain text files, HTML, and source code) requirements. For the long-term, digital text and images are the easiest to digitally archive, readily available in searchable formats (Jung & Zellmann, 2008).

Likewise, digital image files such as photographs, labelled diagrams, and other visual illustrations can provide information rapidly (e.g.,.jpg,.png,.gif, pdf). These can be transmitted and disseminated using a wide variety of readily available devices. For example, mobile devices, emails, and messenger systems are all capable of processing and sharing images (Kress & van Leeuwen, 1996; Mills, 2009; New London Group, 2000). In an academic setting, it is good practice to always include figure headings, legends, and descriptive text alongside any illustrations and images. This means that when used correctly for learning within a tertiary setting, text will often accompany digital photographs providing image-text meaning (Durbin, 2004).

Figures are common within academic writing. In many cases, these incorporate a mixture of text, graphs, images, and illustrations (Liu & Treagust, 2013). It should be noted that depending on the digital text or image type there are varying degrees of scalability, sustainability, and serviceability. For example, specialist software is used to produce or render certain digital illustrations, requiring more than a digital camera, with the need for accompanying descriptive text and figure headings (Franzblau & Chung, 2012).

Text and image formats can also benefit those with disabilities. Text can be easily translated to audio for those who are vision impaired, and digital images can be re-coloured in a way that assists with colour-blindless, or have embedded figure captions, ‘alt text’ or image descriptions (Cain & Fanshawe, 2021). This means that even at scale, this media may be accessible in a way that facilitates a relatively equitable use in education compared to other media.

#### Audio/podcasts

Increasing in popularity is the use of podcasts and audio lessons (Kelly et al., 2022). In academic or professional settings, audio instruction should often be accompanied by a written transcript (Newman et al., 2021), furthering the content’s ability to be scaled and accessible to a broad audience. The most common use of podcasting in higher education is to record lectures or to provide supplementary learning material that students can flexibly view from anywhere at any time for asynchronous learning, unconstrained by time and place (Hew, 2009). One of the main advantages of podcasts for learning is that students can replay missed or difficult information in their own time, and at their own pace.

Audio recordings have an advantage over digital text in relation to being an embodied mode of communication, in which the speaker’s voice creates a direct and personal connection with the listener. Similarly, spoken language use is fundamental to human development, while reading and writing are formalised skills. Although potentially somewhat easy to scale (Zhang et al., 2022), people often struggle with the applications needed to stream podcasts, particularly in a mobile setting. It is for this reason that most students listen to podcasts at home, rather than while on the move (Hew, 2009).

The digital transformation of audio media has seen many rapid technological developments for recording and distributing sound, in the past, moving from superseded cylinder phonographs and nitrate-based film to disc records, cassette, 8-track tape, polyester film, floppy disc, compact disc, and then to mp3, with sound also often transmitted in the context of video, streaming apps, VR, and 360-degree film (Mills et al., 2022b). If the past is an indicator of the future, the technologies of audio recording or podcasting will continue to evolve, having high scalability but limited to a certain extent by sustainability.

#### Animations/moving images

The usefulness of animation may vary depending on the learning and students' learning capacities (Dajani & Abu Hegleh, 2019), however, it is accepted that animation aids learner understanding and is widely used to increase and facilitate students' grasp of challenging practices that vary over time (Ainsworth & VanLabeke, 2004). Although they are primarily moving pictures, updating or editing an animation can be quite prohibitive due to the expertise and time commitment required. In addition, specialist software is often used to create animations, moving images, or kineikonic texts (Mills, 2011), and this can cause issues with both serviceability and sustainability.

A primary example is the discontinuation of Adobe Flash (Adobe Systems Incorporated, San Jose, CA, USA) support on websites, which meant that a large amount of educational content became redundant and inaccessible to the average internet user. This presented challenges for teachers, as the coding for the newer website formats (i.e., HTML5) was entirely different to the skills required to generate content in Adobe Flash (Gluzman & Gorbunova, 2019).

#### Presentation slides

Given the long-term usage of slide presentations in education, whether utilising older technology such as Over Head Transparency or computerised alternatives like PowerPoint, there has also been a distaste among students and instructors for the use of this technology, as the slogan ‘Death by PowerPoint’ attests (Roberts, 2018).

However, slideshows remain the standard provision of information in tertiary instructions, and increasingly in secondary schools, slide presentations are the bread-and-butter form of content delivery in today’s classroom (Bedenlier et al., 2020). However, there are limitations in the ability of PowerPoint slides to scale. Specific software is required to read the files, and embedded content may become unreadable over time as codecs and embedded media readers become obsolete. Presentation slide platforms, such as PowerPoint, also tend to compress the quality of embedded media, making it less than ideal for a sustainable long-term information delivery mode.

To address some of these challenges, cloud-based technologies like Prezi became commonplace in higher education classes (Strasser, 2014). Educators tout Prezi as a more effective medium for information acquisition, however, research has shown that it is not as successful as PowerPoint in long-term learning and retention (Chou et al., 2015). Tools like Prezi are also more difficult to maintain, edit, and learn from, affecting their serviceability. The additional overheads of cloud-based computing also affect the sustainability of these tools as large data centres are required to host the cloud-based artifacts requiring ever-increasing energy requirements. This is also of concern in recent years with increasing governance of data protection, security concerns, and privacy. The other concern is the changing rules by third-party providers from free to paid subscription models and the features provided or not provided by the vendor. This greatly affects long-term serviceability.

#### Video

The use of videos in tertiary education has increased in the past twenty years (Brame, 2016; Fyfield et al., 2019). However, a growing problem where older videos suffer from being recorded at a low resolution, making them blurry and unclear on modern screens. In some cases, changes in resolution do not have an impact on educational outcomes (Plata et al., 2021). However, in fields such as science, low-resolution videos make viewing important features, text, labels, or facets, where clarity and sharpness are required, difficult (Plata et al., 2021). Using technologies and software to upscale older videos can introduce artifacts and errors (Akramullah, 2014), which can be detrimental to learning if fine details are important. With 4 K taking over as the new HD standard, and 8 K televisions available, educators should be wary that video does not necessarily provide any long-term reliable storage or applicability to an educational setting. Video also requires a large amount of storage space, network bandwidth, and does become redundant over time as the presentation quality (e.g., animations, edits, styling, colour corrections) that the community expects increases.

Video can be challenging to edit without appropriate training and software, and much more challenging to update compared to text-based materials. Although social media platforms now focus on video distribution, such as TikTok and Instagram, other older formats are no longer readily available, with the primary example of VINE now archived and videos no longer easily accessible (Summers & Wickner, 2019).

#### Mobile applications

As smartphones in particular are relatively ubiquitous, their use in teaching has become particularly appealing to educators (de Oliveira & Galembeck, 2016). Mobile applications can be incredibly interactive and valuable learning tools (Dsouza et al., 2021), with educators highlighting the benefits and flexibility of mobile applications in the classroom (Arain et al., 2018), especially when compared to their didactic counterparts (Stirling & Birt, 2014).

However, there is often a cost for mobile-based applications, and as students install them on their personal devices, some may be reluctant to download or register for an account, due to privacy concerns. The data privacy risks make some educators, parents, and students cautious in their use of applications installed on their own private devices.

Another concern is redundancy and applications becoming obsolete, developing bugs over time, becoming security risks if not updated, having a recurring annual fee, and as such, becoming far less sustainable. A recent example was the hosting of apps and the change from 32-bit to 64-bit, when older apps were simply deleted from both the Apple and Google Play stores. This is concerning because the stores have only been operational for ten years, during which time apps have become unusable and have even been de-hosted.

At this point of the Triple-S framework, the equity and division between users becomes more apparent. Although mobile devices are relatively ubiquitous, access to high-speed connections, Wi-Fi, data, or even basic service (i.e., rural areas), becomes highly limited in the effectiveness of this as a broad educational media (Lai & Widmar, 2021). This impacts the scalability, where some students may find the mode of media delivery to be more cumbersome, compared to others. For example, if high-resolution video or images are needed, such as with technical applications, the sheer size of the downloadable files may mean that some students would be disadvantaged by the cost of downloads, as well as the accessibility of high-speed connections (Cullinan et al., 2021). This increases the digital divide between the have/have nots, and may not provide an equitable experience at scale (Tate & Warschauer, 2022).

#### Augmented reality

Augmented reality (AR) uses a user’s smartphone, tablet, or digital device’s camera to superimpose digital models into the surrounding real world. These objects can be interacted with on the screen, allowing a fusion between the real and digital spaces (Moro et al., 2021; Moro et al., 2020a). A major benefit of AR is that many students have their own smartphones and there is an increasing accessibility to other smart devices, such as tablets (Mills, 2022). This increases the scalability of the intervention, where AR applications can be distributed relatively easily. However, there are serviceability challenges, such as issues with compatibility (i.e., between iPhone and Android), as well as a requirement to keep the software up to date as devices improve and camera technology changes. As such, for media such as AR to be delivered onto student-owned smart devices, there will need to be expertise and technical support offered by the institution to facilitate this.

Use of the student’s own devices is sustainable, as no new technology needs to be acquired. However, this practice does run the risk of creating an unequitable experience between learners. Older phones may struggle to keep up with the graphical requirements needed to display 3D objects (Moro & Phelps, 2022). Additionally, the technological competence of some students will be more advanced than others, meaning that the progressive style of technology used in AR might be distracting or daunting for those less comfortable with this type of learning. Compared to other immersive media (listed below), AR is currently the easiest to scale and is therefore, likely to continue as the most sustainable, but caution needs to be made when scaling to a diverse cohort of learners.

#### Virtual reality

Virtual reality (VR) technology enables the user to be fully immersed in an artificial environment, which is experienced through sensory stimuli (i.e., sight, hearing, motion) that mimics the properties of the real world through constantly updating, high-resolution head-mounted displays, stereo headphones and motion-tracking systems (Moro et al., 2020b). While there are clear benefits of VR over other technology for creating situated learning that simulates skills that would be dangerous to perform or impossible to experience in the real world (Mills et al., 2022a), the use of a head-mounted display creates challenges for scaling and servicing. VR requires constant updating of software, as well as regular updating of the device itself as older versions become redundant. In addition, only one user can utilise VR at a time, which makes this delivery mode less scalable than many other instructional formats. Research suggests that VR technologies are currently experiencing high growth in the education sector (Elmqaddem, 2019), suggesting that the scalability, sustainability, and serviceability will improve in future decades.

The rapid advancement in VR technology over recent years has required universities to invest in regular upgrades and updates to hardware. In particular, where VR is used in class and students need to be present on-campus to utilise it. The included circuitry within the VR computing systems, difficulties in recycling, and the large nature of the devices means that the technology has likely contributed to a growing amount of e-waste, impacting the environmental sustainability of this technology over the long term.

#### Mixed reality

Using head-mounted displays, such as smart glasses (e.g., Microsoft HoloLens, Google Glass, Vuzix Blade), mixed reality (MR) merges the real environment with augmented components, responding to the user’s gestural cues, voice, and gaze. Smart glasses differ from VR with a see-through optical display so that the real world is visible, rather than blocked from view, and devices have built-in processors allowing for mobile use (Nichols & Jackson, 2022). Mixed reality headsets, including augmented reality smart glasses (Ro et al., 2018), are currently expensive, impacting the overall scalability. The novelty of this developing technology will also make it challenging to hold confidence in providing lessons that are sustainable or useable over the long term. The use of these devices in providing educational content across different concepts and levels of education is still largely emergent, particularly for schooling (Maas & Hughes, 2020).

Mixed reality is one of the fastest growing technologies this decade, with the potential for wide utilisation (Southgate et al., 2016), particularly as entire worlds, such as the metaverse, come to fruition. As such, although mixed reality sits as the least scalable, sustainable, and serviceable media presented in these examples, this is likely to improve over time as this technology becomes more accessible.

## Conclusions

Although the recommendation so far has been to assess the viability of individual media provision using the Triple-S framework, the challenges for scalability, sustainability, and serviceability can be confounded by the delivery media. A large amount of content is now provided within Learning Management Systems (LMS). Commonly used formats in universities include Blackboard Learn, Moodle, Canvas, Edmodo, and Absorb LMS. Although LMS represent the main mode for content delivery within tertiary institutions, they remain proprietary, challenging to scale, and have difficulties with compatibility between institutions and external software. This means that content created within a LMS may end up redundant after an update, change of service provider, or alteration in functionality. This highlights that beyond the Triple-S framework, how the desired content is delivered to students can also offer a confounding factor regarding its scalability, sustainability, and serviceability.

Websites are another commonly-used delivery mode, and with the introduction of smartphones, websites can now be accessed by students at any time of the day (Mills, 2015a). Creating a public website does require an additional ‘click-on-a-link’ for students, but can provide content in a consistent way anywhere in the world (Alajarmeh, 2022). The functionality of websites can change and have significant consequences in the long term, and as such, if the media (e.g., video, illustration) is dependent upon a web delivery, this then needs to be considered as a contributing factor that could impact the long-term viability of the intervention.

Although the Triple-S framework has identified specific media as examples, it is relatively easy to use it to consider other interventions. For example, interactive live online polling in class (e.g., Kahoot!) would sit at the same step as mobile applications (Phelps & Moro, 2022). There is usually a recurring fee charged to the university for the software’s use, and problems with accounts (such as students being unable to log in) may cause serviceability issues. This would be the same case with the introduction of serious games (Moro et al., 2022b) or digital simulations, sitting approximately at the level of mobile applications on the framework exemplar (Fig. 3).

This article has introduced the Triple-S framework, a scale implemented to assist educators in decision-making around the procurement or implementation of digital devices or software. Although technology advancements have provided educators with a wealth of options to choose from for content delivery, it is important to assess the scalability, sustainability, and serviceability prior to any implementation. As digital options for content delivery and class interactions become more advanced and novel, the intervention often becomes less scalable, sustainable, and serviceable. As such, any discussions about novel technological implementations into a program could benefit from outlining the expectations around the Triple-S framework.

Modern educators have ample choice when procuring novel teaching and learning technology, and for the delivery of content through technology-enhanced media. In many cases, this provides great benefit. For example, text is certainly not the best way to present three-dimensional organs in a medical course, and as such, educators may wish to utilise interactive websites or augmented and virtual reality presentations. However, when pursuing this option, curricula developers also need to be aware that this practice will result in their material becoming less scalable, less sustainable, and less serviceable. As such, consideration of the Triple-S framework should not discourage educators from pursuing higher-level media or technology, but aims solely to provide concepts to consider when investigating the positives and negatives of each intervention. It is envisioned that prior to any acquisition of new technology, or consideration of novel delivery modes for educational content, a discussion regarding its position on the Triple-S framework is conducted.

## Data Availability

Any datasets used and/or analysed during the current study are available from the corresponding author on reasonable request.
